# Maintenance of thermogenic adipose tissues despite loss of the H3K27 acetyltransferases p300 or CBP

**DOI:** 10.1152/ajpendo.00120.2024

**Published:** 2024-08-14

**Authors:** Daniel Gamu, Makenna S. Cameron, William T. Gibson

**Affiliations:** ^1^School of Kinesiology, University of British Columbia, Vancouver, British Columbia, Canada; ^2^Department of Medical Genetics, University of British Columbia, Vancouver, British Columbia, Canada; ^3^BC Children’s Hospital Research Institute, Vancouver, British Columbia, Canada

**Keywords:** brown adipose, histone acetyltransferase, metabolism, obesity, thermogenesis

## Abstract

Brown and beige adipose tissues are specialized for thermogenesis and are important for energy balance in mice. Mounting evidence suggests that chromatin-modifying enzymes are integral for the development, maintenance, and functioning of thermogenic adipocytes. p300 and cAMP-response element binding protein (CREB)-binding protein (CBP) are histone acetyltransferases (HATs) responsible for writing the transcriptionally activating mark H3K27ac. Despite their homology, p300 and CBP do have unique tissue- and context-dependent roles, which have yet to be examined in brown and beige adipocytes specifically. We assessed the requirement of p300 or CBP in thermogenic fat using uncoupling protein 1 (*Ucp1*)*-*Cre-mediated knockdown in mice to determine whether their loss impacted tissue development, susceptibility to diet-induced obesity, and response to pharmacological induction via β_3_-agonism. Despite successful knockdown, brown adipose tissue mass and expression of thermogenic markers were unaffected by loss of either HAT. As such, knockout mice developed a comparable degree of diet-induced obesity and glucose intolerance to that of floxed controls. Furthermore, “browning” of white adipose tissue by the β_3_-adrenergic agonist CL-316,243 remained largely intact in knockout mice. Although p300 and CBP have nonoverlapping roles in other tissues, our results indicate that they are individually dispensable within thermogenic fats specifically, possibly due to functional compensation by one another.

**NEW & NOTEWORTHY** The role of transcriptionally activating H3K27ac epigenetic mark has yet to be examined in mouse thermogenic fats specifically, which we achieved here via *Ucp1*-Cre-driven knockdown of the histone acetyltransferases (HAT) p300 or CBP under several metabolic contexts. Despite successful knockdown of either HAT, brown adipose tissue was maintained at room temperature. As such, knockout mice were indistinguishable to controls when fed an obesogenic diet or when given a β_3_-adrenergic receptor agonist to induce browning of white fat. Unlike other tissues, thermogenic fats are resilient to p300 or CBP ablation, likely due to sufficient functional overlap between them.

## Graphical abstract

**Figure:**
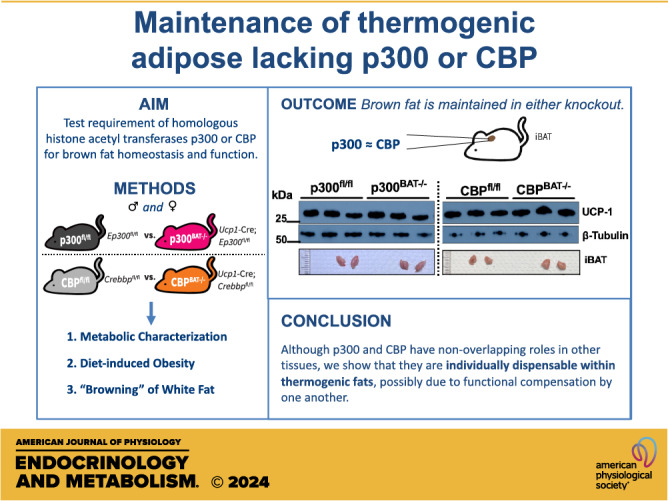

## INTRODUCTION

Brown adipose tissue (BAT) is specialized for thermogenesis because it contains uncoupling protein (UCP)-1 within its mitochondrial inner membrane. When activated by the sympathetic nervous system, UCP-1 uncouples ATP production from the mitochondrial electrochemical H^+^ gradient, creating heat instead of ATP (1). As such, BAT activity is an integral component of mammalian adaptive nonshivering thermogenesis. In addition to discrete depots of classical brown fat, beige (or “brite”) adipocytes represent a distinct UCP-1-positive cell type interspersed within subcutaneous white fat (2–5), the appearance of which (i.e., “browning”) can be induced physiologically or pharmacologically (e.g., cold and adrenergic agonists) (6–9).

Numerous rodent studies have shown that altering brown/beige fat thermogenesis impacts energy balance and susceptibility to obesity, particularly when housed at room temperature. As such, there has been considerable historical interest in harnessing BAT activity as a means to reduce body weight and improve systemic metabolism (10), particularly given that BAT activity correlates with lower cardiometabolic risk factors (11–13). It should also be noted that brown fat is more than a thermogenic organ. Like white fat, BAT secretes numerous peptides and metabolites with paracrine and endocrine function termed BATokines (12). Such signals may help mitigate various pathophysiological features accompanying obesity and type 2 diabetes, independent of thermogenesis. Despite the expanding role brown fat plays in metabolism, our understanding of pathways governing its development, adaptability, and lifespan is incompletely understood. It has become increasingly clear over the past decade that mechanisms regulating chromatin structure are vital for controlling the plasticity of thermogenic adipocytes.

Within chromatin, DNA is wrapped around histones (H), which are important for nuclear DNA packaging and gene expression. Posttranslational modifications (PTMs) of histones tighten or loosen chromatin’s conformation, thereby controlling the transcriptional machinery’s access to important regulatory sites. One histone residue critical for regulating gene expression is H3 lysine 27 (i.e., H3K27); this site is either acetylated (H3K27ac) or mono/di/trimethylated (H3K27me1-3), modifications that are associated with transcriptional activation or repression, respectively (14). Inhibiting various histone deacetylases (HDACs), the enzymes that remove acetyl moieties, can improve thermogenic functioning of brown/beige fat and mitigate metabolic dysfunction associated with obesity (15–21). The metabolic benefits associated with HDAC loss-of-function are presumed to result from greater occupancy by the transcriptionally activating H3K27ac mark at critical loci controlling BAT thermogenesis. Although studies of HDACs suggest that H3K27ac is a critical molecular switch controlling BAT identity and function, they have failed to consider the histone acetyltransferases (HATs) that actively lay down the H3K27ac mark.

Acetylation of H3K27 depends on the activity of CREB-binding protein (CBP) and its functional homologue p300 (22, 23). Owing to the large number of signaling proteins with which they interact (24), these ubiquitously expressed HATs fulfill broad-ranging cellular functions. Although numerous classes of histone acetyltransferases exist (25), p300/CBP are solely responsible for catalyzing H3K27ac formation (26–28). Based on murine loss-of-function studies, the role p300/CBP play in tissue development, metabolism, and energy balance is complex. With respect to CBP, whole body heterozygous null mice develop lipodystrophy and are resistant to diet-induced obesity, but are paradoxically insulin sensitive (29). Conversely, hypothalamic-specific knockout of CBP causes obesity and impaired glucose homeostasis and decreases thermogenesis (30). Furthermore, p300 and CBP do not always display functional redundancy. For example, during mouse embryogenesis, p300 but not CBP is required for myogenic differentiation (31), whereas knockout of CBP using adiponectin-*Cre* markedly alters gene expression programs in an adipose depot-specific manner, whereas p300 does not (32). These studies highlight the need to study each HAT individually in a tissue- and context-dependent manner.

Evidence for a role of p300/CBP in thermogenic adipocytes is currently indirect. For example, adrenergic stimulation of BAT with isoproterenol increases binding of p300/CBP and concomitant H3K27ac deposition at thermogenic genes, including *Ucp1* and *Pgc1a* (19, 33). Similarly, HAT binding and H3K27ac enrichment at the *Prdm16* locus, a protein critical for maintaining brown fat identity (34), increases as brown adipocytes mature in vitro (35). Together, these studies suggest that transcriptional activation of gene programs integral for BAT development and thermogenesis requires adequate H3K27ac levels; however, it is yet unclear whether this relationship is obligatory, and if so, to what extent. Here, we sought to determine the in vivo requirement of p300 or CBP specifically in the maintenance and remodeling of thermogenic adipose tissues. We hypothesized that lack of either histone acetyltransferase would prevent transcriptional activation of thermogenic programming, thereby impairing brown and beige fat development, adaptability, and energy balance in mice.

## MATERIALS AND METHODS

### Animals

Mice carrying *Cre-recombinase* driven by the *Ucp1* promoter (i.e., *Ucp1-Cre*; Strain No. 024670; B6.FVB-Tg(Ucp1-cre)1Evdr/J) and those with *loxP* sites flanking exon 9 (E9) of *Crebbp* (Strain No. 025178; B6.Cg-Crebbptm1Jvd/J) were purchased from Jackson Laboratories. Mice with *loxP* sites flanking E9 of *Ep300* were generously provided by Dr. Paul Brindle (St. Jude Children’s Research Hospital, Memphis, TN) and have been described previously (24). Breeding pairs consisted of mice that were homozygous floxed for either the *Ep300* or *Crebbp* alleles (i.e., *Ep300*^fl/fl^ or *Crebbp*^fl/fl^); male hemizygous carriers of *Ucp1*-*Cre* were bred with *Cre*-negative females, generating floxed controls (i.e., p300^fl/fl^ or CBP^fl/fl^) and BAT-specific knockout mice (i.e., p300^BAT−/−^ or CBP^BAT−/−^). In addition, all mice were homozygous for the dual-fluorescence *Cre*-reporter allele ROSA^mT/mG^ (Jackson Laboratory; Strain No. 007576); thus, cell membrane-localized tdTomato (mT) is replaced by membrane-localized green fluorescent protein (mG) in *Cre*-*recombinase*-expressing cells only. All animals were housed at room temperature (∼22°C), provided water and standard rodent chow (Teklad-2918) ad libitum under a 12-h light/12-h dark cycle at BC Children’s Hospital Research Institute animal facility. Experiments were conducted with 10- to 12-wk-old male and female mice and were approved by the University of British Columbia Animal Care Committee in accordance with the Canadian Council on Animal Care guidelines.

### Metabolic Phenotyping

Whole body energy expenditure, O_2_ consumption, CO_2_ production, respiratory exchange ratio (RER) [carbon dioxide consumption (V̇co_2_)/oxygen consumption (V̇o_2_)], spontaneous cage activity, and food and water consumption were measured at room temperature in the PhenoMaster home cage metabolic platform (TSE Systems). Data acquisition was recorded over 96 h, with the first 24 h discarded to remove the effects of acute handling stress on mice. Following metabolic cage experiments, acute cold tolerance was examined by lowering housing temperature to 4°C for 6 h. Immediately before and following the cold challenge, intrarectal temperature was measured with a RET-3 thermocouple probe (Physitemp) and digital thermometer (ThermoWorks). Mice were then housed back at room temperature for 1 wk, after which they were anesthetized with isoflurane and euthanized by cervical dislocation. Both lobes of interscapular brown fat were then dissected and cleared of extraneous connective tissue and muscle, individually snap frozen in liquid nitrogen, and stored at −80°C until analyzed (analysis described in *qPCR and Western Blotting*). Contralateral inguinal white fat depots were harvested along the entire inguinal crease of each mouse and individually snap frozen as earlier following the removal of lymph nodes.

### Diet-Induced Obesity

Beginning at 10–12 wk of age, group-housed mice were provided ad libitum access to water and a “Westernized” high-fat diet (HFD) (TD.88137, Envigo; 42% kcal fat, 48.5% kcal carbohydrate, 15.2% kcal protein) for 16 wk as we have previously done (36–39). Fresh food was presented every other day, with animal body mass recorded weekly. Body composition was measured in nonanesthetized mice by quantitative magnetic resonance (EchoMRI-100H, Echo Medical Systems) before and after 8 and 16 wk of high-fat feeding. At these same timepoints, whole body glucose tolerance was analyzed following a 5-h fast; mice were given an intraperitoneal injection of glucose (2 g/kg body mass), with blood glucose (OneTouch Ultra; Johnson and Johnson) sampled via tail prick at 0, 15, 30, 60, and 90 min after injection.

### Animal Remodeling of Thermogenic Adipose Tissue by a Selective β3-Receptor Agonist

To induce beige adipogenesis (i.e., “browning” of subcutaneous white fat), mice were given the highly selective β_3_-adrenergic receptor (AR) agonist CL-316,243 (CL; Sigma; 1 mg/kg body mass) dissolved in saline. Mice were weighed and given an intraperitoneal injection of CL or a weight-adjusted volume of vehicle control daily for 7 consecutive days. Twenty-four hours following the final injection, mice were euthanized, and adipose tissues were collected as described in *Metabolic Phenotyping*.

### qPCR and Western Blotting

One unit of snap-frozen adipose tissue (i.e., interscapular lobe or inguinal depot) was homogenized in TRIzol using a bead mill (Bullet Blender, Next Advance) and 0.5-mm zirconium oxide beads. RNA was then column purified (RNeasy Mini Kit, Qiagen) following on-column DNase digestion (Qiagen, Cat. No. 79254) according to the manufacturer’s instructions. About 1 µg of total RNA was reverse-transcribed (SuperScript III: Invitrogen, Cat. No. 18080-051) using oligo(dT)_20_ primers, after which cDNA was stored at −20°C. On the day of use, cDNA was diluted 1:10 in nuclease-free H_2_O, with adipose transcripts amplified with GoTaq qPCR master mix (Promega, Cat. No. A6001) using a ViiA 7 PCR system (Applied Biosystems) set to standard cycling conditions. Gene expression data were normalized to *Actb* or *Ppia* and calculated using the 2^−ΔΔCt^ method. Primer pairs are listed in Table 1.

**Table 1.** qPCR primer sets

Gene	Forward (5′–3′)	Reverse (5′–3′)
*Ep300* E9	CAAGCCATATTTCCCACTCCG	TGCAGATTCATACATGTCCCCT
*Crebbp* E9	TTTCTCTGCTAATAAATGATAGTATTCATC	CTCGTTCAAGCCATCTTCCC
*Ucp1*	GGACGACCCCTAATCTAATG	GACGTCATCTGCCAGTATT
*Pgc1a*	ATGCAGACCTAGATACCAACT	CCATCTCTCTGTCATTCCTC
*Dio2*	AGTCTTTTTCTCCAACTGCC	CCCAGTTTAACCTGTTTGTAGG
*Elovl3*	TTGTTGAACTGGGAGACAC	TACATGACAGAATGGACGC
*Pparg*	GGACTGTGTGACAGACAAGATTT	AATCAACTGTGGTAAAGGGC
*Fabp4*	CTGGTGGTGGAATGTGTTAT	CCGACTGACTATTGTAGTGT
*Adipoq*	ACGTTACTACAACTGAAGAGC	GGACCAAGAAGACCTGCAT
*Crebbp*	AGACCCTGCAGCTCTGAAAGATC	TGTCTCCCTCCACTTTCTTAGCA
*Ep300*	CATCTGGGTTTGTCTGTGA	CTCGATTCTCCAGAAAGGTC
*Actb*	TCCTTCTTGGGTATGGAATCCTG	TGGCATAGAGGTCTTTACGGA
*Ppia*	CAAGACTGAATGGCTGGATG	CCTGAGCTACAGAAGGAATG

*Ucp1*, uncoupling protein 1.

A second sample of snap-frozen adipose tissue was homogenized in RIPA buffer supplemented with Halt protease/phosphatase inhibitor cocktail (Prod. No. 1861284, ThermoScientific) using a bead mill, including the histone deacetylase inhibitor sodium butyrate (10 mM final concentration). Protein concentration was determined by the bicinchoninic acid (BCA) assay (ThermoScientific) using bovine serum albumin as standards. Following electrophoresis, samples were wet-transferred onto PVDF membranes (pore size: 0.2 µm) and probed overnight at 4°C for UCP-1 (ab10983, Abcam), mitochondrial respiratory chain subunits (total OXPHOS; ab110413, Abcam), and for the following using primary antibodies from Cell Signaling Technologies: green fluorescent protein (GFP; 4B10), acetylated H3K27 (Lys27), H3 (D1H2), and β-tubulin (D3U1W). Following incubation in appropriate secondary antibodies (Cell Signaling Technology), immunoblots were visualized on film using enhanced chemiluminescent substrates. Immunoblots were then incubated in Ponceau stain (BioShop), and densitometry analyses were performed using ImageJ software.

### Statistics

Mean differences between two groups were analyzed by Student’s *t* test (two-tailed, independent samples), whereas all other data were analyzed by two-way ANOVA or two-way repeated-measures ANOVA, followed by a Holm–Šídák post hoc comparisons where appropriate (GraphPad Prism 10.0.0). Statistical significance was considered at *P* < 0.05. Values are presented as means ± SE.

## RESULTS

### Metabolic Characterization of Knockout Mice

**Figure 1.**
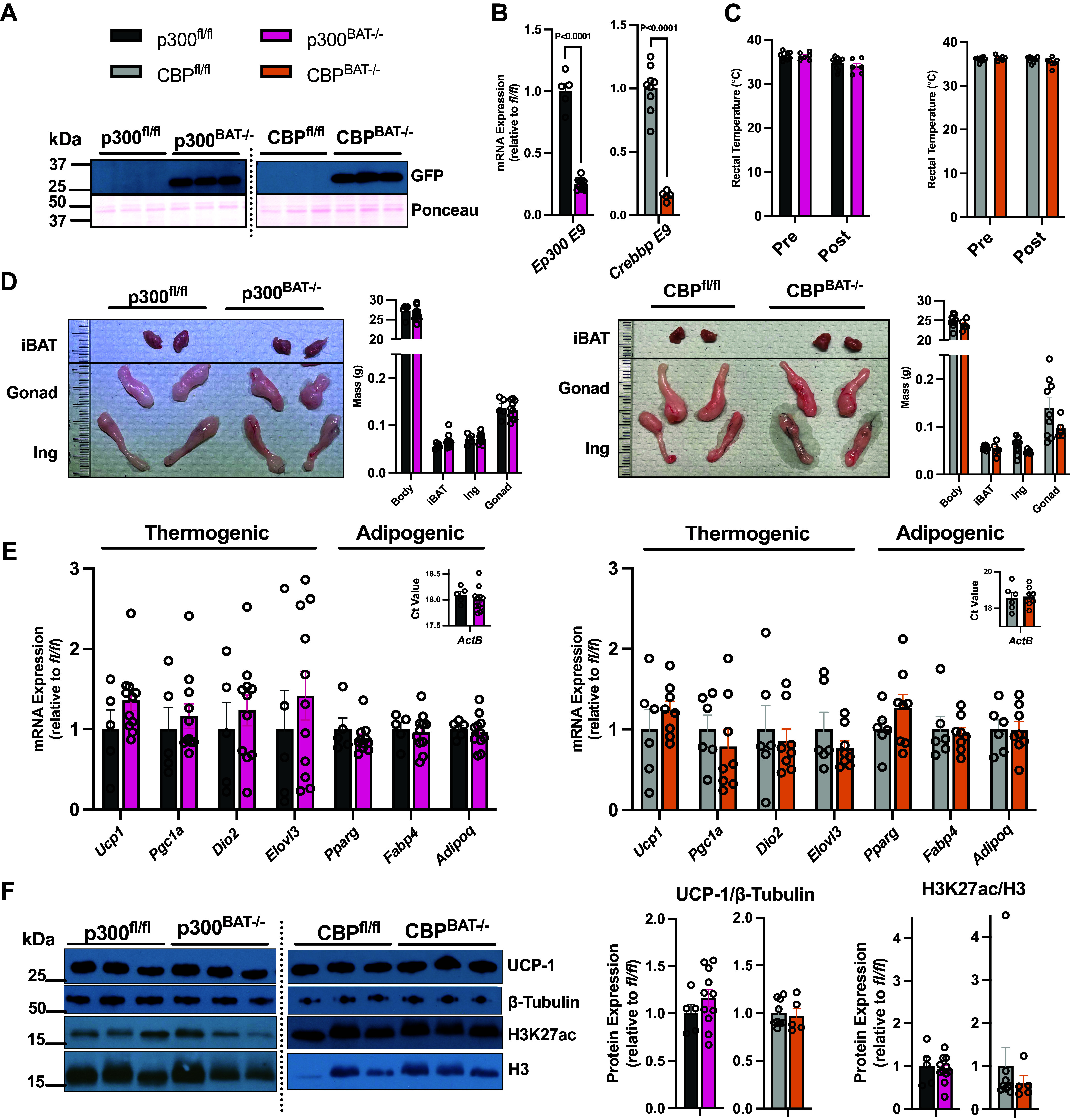
Loss of p300 or CREB-binding protein (CBP) does not impair brown adipose tissue (BAT) formation or acute cold tolerance of male mice in vivo. *A*: positive expression of green fluorescent protein in p300^BAT−/−^ and CBP^BAT−/−^ only. Equal protein load was confirmed by Ponceau stain. *B*: knockdown of brown fat *Ep300* and *Crebbp* exon 9 gene expression. *C*: intrarectal temperature (°C) before (pre) and after (post) exposure to 4°C for 6 h. *D*: body mass and tissue wet weights (g) of interscapular brown fat (iBAT), inguinal (Ing), and gonadal (Gonad) white adipose depots with representative photographs. *E*: brown adipose tissue thermogenic and adipogenic marker gene expression. Transcripts were normalized to *Actb* and expressed relative to *fl/fl* controls. *Inset*: Ct values for *Actb*. *F*: interscapular brown adipose tissue protein expression. Values are presented as Avg. ± SE. *n* = 5–10/group.

Successful *Cre*-mediated recombination in brown fat was noted by a marked induction of GFP content and concomitant knockdown of *Ep300* and *Crebbp* exon 9 gene expression in p300^BAT−/−^ and CBP^BAT−/−^ mice of either sex (Fig. 1, *A* and *B*; Supplemental Fig. S1, *A* and *B*). Despite HAT depletion from within brown fat, whole body energy expenditure and substrate oxidation preference (RER) were comparable at room temperature between control and knockout mice (Supplemental Fig. S2*A*). Furthermore, feeding/drinking behavior and spontaneous cage locomotion did not differ between knockout mice and their respective floxed littermate controls (Supplemental Fig. S2, *B*–*D*). Although not a specific measure of brown fat’s maximal capacity for nonshivering thermogenesis, we next assessed acute cold tolerance by measuring intrarectal temperature following exposure to 4°C, which knockout mice were able to withstand without developing relative hypothermia after 6 h of exposure (Fig. 1*C*; Supplemental Fig. S1*C*).

Following our whole body metabolic characterization, adipose tissues were excised for examination of molecular markers of thermogenesis and adipose development. Several studies deleting histone-modifying proteins via *Ucp1-Cre* have shown altered gross morphology of brown fat and gene programming of chow-fed mice at room temperature (40, 41). Given their broad-reaching roles, we expected that knockout of either HAT would impact brown fat mass and gene programming. However, unlike hypothesized, loss of p300 or CBP did not impair brown fat formation, as noted by comparable interscapular BAT (iBAT) wet weights (Fig. 1*D*; Supplemental Fig. S1*D*). In addition, white adipose tissue (WAT) depot masses were mostly unaffected by p300 or CBP knockout, further suggesting energy balance was not perturbed in these mice (Fig. 1*D*; Supplemental Fig. S1*D*). Female CBP^BAT−/−^ mice had less inguinal WAT but no changes in gonadal WAT (Supplemental Fig. S1*D*). Surprisingly, we found no impairment in the expression of thermogenic and adipogenic marker genes despite loss of either of these major transcriptional coactivators (Fig. 1*E*; Supplemental Fig. S1*E*). *Pparg* expression was higher in female CBP knockout mice, but there were no other significant changes in the gene expression (Supplemental Fig. S1*E*). Furthermore, iBAT UCP-1 protein content and the proportion of H3 that were detectable as global H3K27ac remained unchanged by p300 or CBP deletion, irrespective of sex (Fig. 1*F*; Supplemental Fig. S1F).

### Diet-Induced Obesity

**Figure 2.**
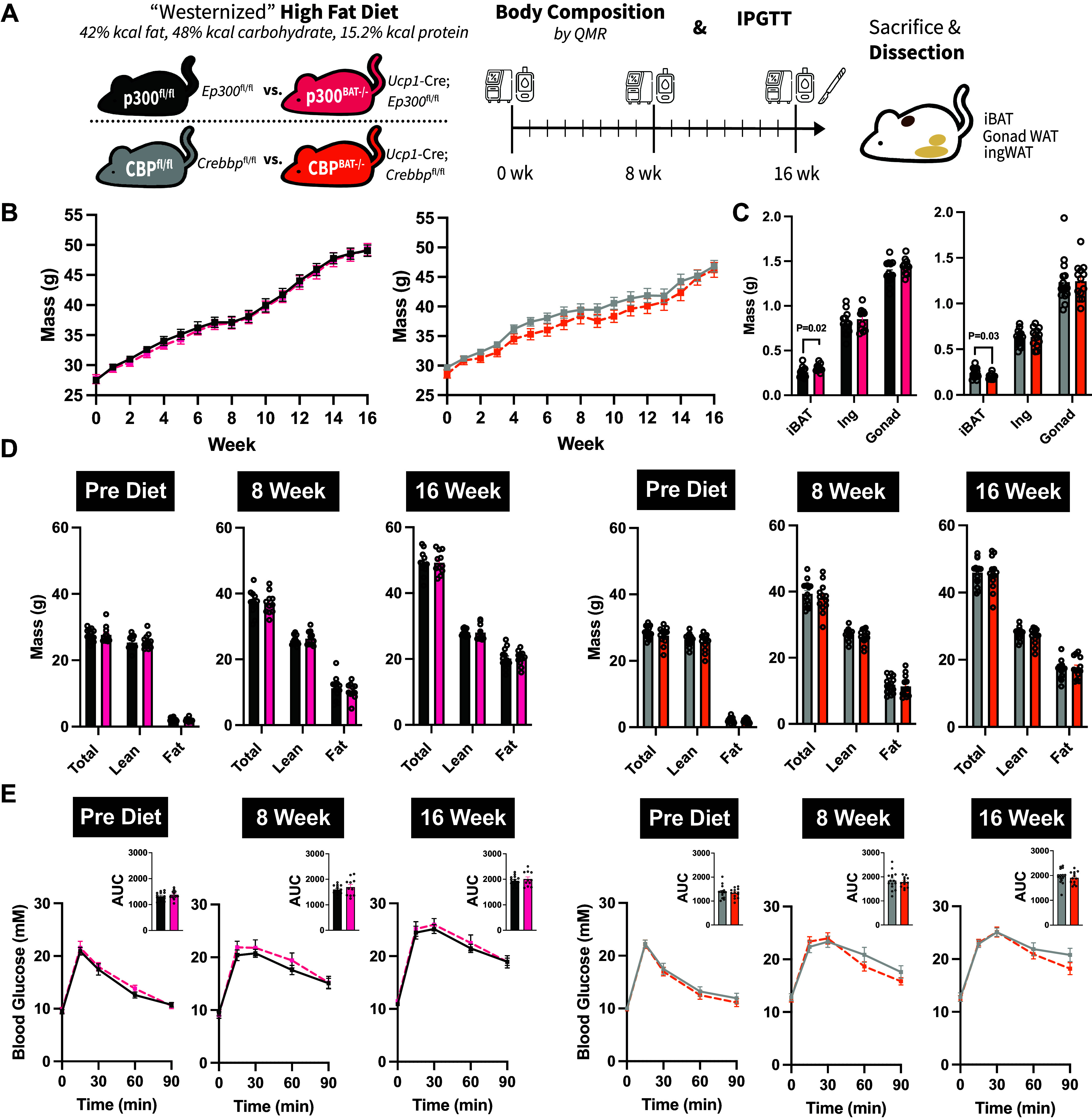
Brown adipose tissue (BAT)-specific loss of p300 or CREB-binding protein (CBP) does not predispose male mice to diet-induced obesity. *A*: experimental design of 16-wk high-fat diet with 3 measurements of body composition and intraperitoneal glucose tolerance tests (IPGTT). *B*: body mass (g) throughout the course of the 16-wk “Westernized” high-fat diet. *C*: tissue wet weights (g) of interscapular brown fat (iBAT), inguinal (Ing), and gonadal (Gonad) white adipose depots following 16 wks of high-fat diet. *D*: body composition (total, lean, and fat mass) by quantitative magnetic resonance (QMR) immediately before (prediet) and at 8 and 16 wk of high-fat feeding. *E*: intraperitoneal glucose tolerance of mice throughout the dietary course. *Inset*: area under the glucose curve (AUC). Values are presented as Avg. ± SE. *n* = 7–15/group.

Although integral for adaptive thermogenesis, brown adipose tissue plays other roles in regulating peripheral metabolism via production and release of BATokines, some of which are important for regulating fatty acid oxidation and glucose metabolism (42–45), among other functions. Furthermore, BAT thermogenesis can be modulated by calorie surfeit in rodents (i.e., diet-induced thermogenesis) (1, 46). Given their role in coordinating transcriptional activation of gene programs, we next determined whether p300 or CBP activity was obligatory in the context of diet-induced metabolic programming of brown fat. To do so, mice were given a “Westernized” high-fat diet (HFD) for 16 wk to induce obesity and impair glucose handling (Fig. 2*A*). Weight gain was comparable between floxed and knockout mice of either sex throughout the HFD (Fig. 2*B*; Supplemental Fig. S3*B*). Consistently, body composition (i.e., total/lean/fat mass) was comparable between genotypes immediately before, after 8 wk, and after 16 wk of diet-induced obesity, suggesting diet-induced thermogenesis remained intact in knockout mice (Fig. 2*D*; Supplemental Fig. S3*D*).

Despite the similar degree of obesity, high-fat feeding had disparate effects on iBAT mass, specifically in male p300 and CBP knockouts. Compared with controls, iBAT wet weight was heavier after HFD in p300^BAT−/−^ mice, whereas the opposite was found in CBP^BAT−/−^ (Fig. 2*C*). Despite these findings, no differences were found in WAT depot weights (Fig. 2*C*; Supplemental Fig. S3*C*). Furthermore, glucose tolerance was not different between control and knockout mice before and throughout the HFD (Fig. 2*E*; Supplemental Fig. S3*E*).

### “Browning” of White Adipose Tissue by a Selective β_3_-Adrenergic Receptor Agonist

**Figure 3.**
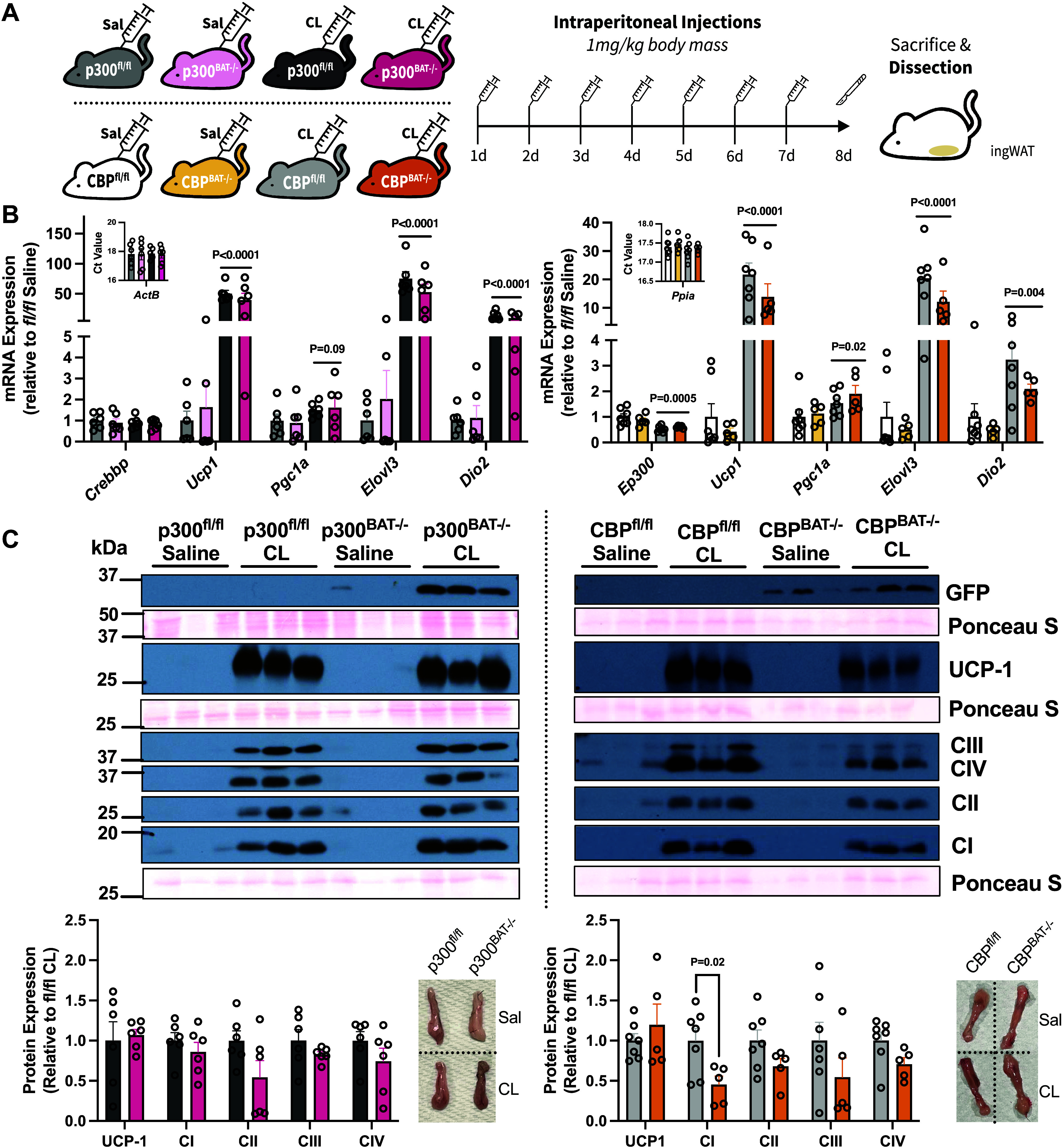
“Browning” of subcutaneous white fat in response to a β_3_-adrenergic receptor agonist is unaffected by p300 or CREB-binding protein (CBP) loss in male mice. *A*: experimental design of β-agonist study with daily intraperitoneal injections for 7 days. *B*: expression of thermogenic marker genes and H3K27 acetyltransferases of subcutaneous inguinal white adipose fat. Transcripts were normalized to *Actb* or *Ppia* and expressed relative to saline treated fl/fl controls. *Inset*: Ct values for *Actb* or *Ppia*. *C*: inguinal fat protein expression of saline- and CL-treated mice. Proteins were normalized to their corresponding Ponceau S stains below. Values are presented as Avg. ± SE. *n* = 5–7/group.

Signaling through the β_3_ isoform of the adrenergic receptor (AR) is critical for acutely activating BAT and for enhancing its thermogenic capacity in mice (1). Furthermore, β_3_-AR agonism stimulates the appearance of beige adipocytes within subcutaneous fat (i.e., “browning” of white fat). Although our previous experiments revealed p300 and CBP to be dispensable within classical brown adipose tissue, we next sought to determine whether they were required for the induction of adipose “browning” in vivo, specifically because beige adipocytes arise from a distinct cellular lineage than that of brown adipocytes. To test whether either HAT coordinates this phenomenon, mice were given daily intraperitoneal injections of the highly selective β_3_-AR agonist CL-316,243 or saline vehicle for 7 days (Fig. 3*A*). As expected, GFP expression was markedly induced in CL-treated knockout mice only (Fig. 3*C*; Supplemental Fig. S4*C*), indicating successful recombination within newly formed beige adipocytes specifically. Expression of thermogenic genes was markedly induced by CL treatment, but were unaffected by mouse genotype (Fig. 3*B*; Supplemental Fig. S4*B*). UCP-1 and mitochondrial respiratory chain complex protein expression were completely absent or nearly undetectable in saline-treated inguinal (ING) depots, but were greatly enhanced by CL administration (Fig. 3*C*; Supplemental Fig. S4C). Given the inconsistency of mitochondrial protein content of saline controls, we chose to compare their expression in CL-treated mice only. Consistent with our gene expression findings, UCP-1 protein and respiratory chain components were expressed equivalently between control and knockout mice, suggesting adipose “browning” remained intact despite loss either H3K27 acetyltransferase. Interestingly, CL-treated p300^BAT−/−^ females had lower expression of complex IV, whereas CBP^BAT−/−^ males and females expressed lower levels of complex I following β_3_-agonism (Fig. 3*C*; Supplemental Fig. S4*C*).

## DISCUSSION

Previous studies of histone deacetylases have suggested that the transcriptionally activating H3K27ac mark is critical for functional homeostasis and adaptability of both brown and beige adipose tissues (15–21). Here, we tested the hypothesis that p300 and CBP, two epigenetic writers that catalyze H3K27ac formation, would each be required for brown and beige adipose programming in vivo. Using *Ucp1-Cre* to selectively delete these HATS from thermogenic adipocytes (leaving white fat intact), we show that p300^BAT−/−^ and CBP^BAT−/−^ mice retained their ability to form and maintain classical brown fat mass and functionality. As such, knockout mice were not susceptible to diet-induced obesity or dysregulation of whole body glucose handling. Furthermore, the absence of p300 or CBP did not prevent the appearance of beige adipocytes within the inguinal depot (i.e., “browning” of white fat). Together, our findings indicate that the p300 and CBP are individually dispensable for thermogenic gene programming of murine brown and beige fats.

Unlike other adipose-selective Cre systems (e.g., *Adipoq*-*Cre* and *Fabp4*-*Cre*) (47), we chose *Ucp1*-*Cre* for our studies because it would target recombination within brown and beige adipocytes specifically (48). Furthermore, it has been used to create viable BAT-specific knockouts of several histone-modifying enzymes (16, 40, 41, 49). Because *Ucp1* expression is detected abundantly in late fetal development (i.e., at E19) and maintained into adulthood (50), we expected that knockout of H3K27 acetyltransferases to markedly impair the developmental expansion of brown fat, resulting in a persistent impairment of thermogenic function into adulthood of knockout mice; such a phenotypic trajectory has been observed when knocking out other epigenetic regulators important for transcriptional activation with *Ucp1-Cre* (16, 40, 41). Given their role in chromatin relaxation, we predicted the loss of p300/CBP to inhibit thermogenic gene programming in BAT. Despite successful knockdown, p300^BAT−/−^ and CBP^BAT−/−^ mice showed no change in whole body energy metabolism, expression of thermogenic marker genes and proteins (e.g., UCP-1), or acute cold tolerance. It is worth noting that shivering is the primary source of thermogenesis in response to acute cold exposure (1). However, BAT-derived nonshivering thermogenesis is still recruited in this context (46, 51), albeit not maximally. However, given that thermogenic gene programming was unperturbed in our knockout mice, it is unlikely that a subtle effect of HAT deletion on acute thermoregulation is being missed in this context.

Despite the lack of a major H3K27 acetyltransferase, p300^BAT−/−^ and CBP^BAT−/−^ mice fully retained global H3K27ac levels of BAT, as measured by Western blot. An important limitation of our methodology here is that we cannot infer whether site-specific enrichment of H3K27ac across various loci is unaffected by knockout of either p300 or CBP from brown fat. Given the multitude of histone modifications important for regulating chromatin structure and gene expression, it is possible that gene-specific acetylation may be depleted in either knockout with minimal effect on the brown fat phenotype. Although this may suggest that either acetyltransferase plays a minimal role in the homeostasis of BAT, we believe that the absence of a phenotype in our mice is more likely due to functional compensation by the other. In support of this, single-gene knockout of either p300 or CBP from mouse embryonic fibroblasts is inconsequential to global H3K27ac content, whereas depletion of both drastically reduces H3K27ac (28). Similar to our findings here, tissue-specific studies of p300 and CBP in skeletal muscle (52–55) and various adipose tissues (32) have found their individual absence inconsequential to tissue homeostasis and function, whereas double-knockout causes pronounced and rapid-onset phenotypes. Thus, the presence of just one homologue likely provides sufficient functional capacity to maintain gene programming and preserve brown fat development and metabolism.

Brown adipose metabolism can be altered by various conditions including prolonged high-fat feeding, which increases UCP-1 expression (1, 36). Furthermore, numerous rodent studies have shown that impairing brown adipose tissue metabolism leads to obesity and metabolic dysfunction in room temperature-housed mice. Although our initial experiments revealed no major defects in knockout mice, we could not rule out a contextual requirement of p300 or CBP activity, particularly during states known to remodel brown fat metabolism, thus requiring transcriptional activation of adaptive gene programs. As such, mice were fed an obesogenic diet to see if a phenotype would emerge during prolonged calorie surfeit. Consistent with our initial metabolic characterizations, knockout mice were not susceptible to diet-induced obesity or dysregulated glucose handling as hypothesized, suggesting diet-induced recruitment of UCP-1 and energy balance were unaffected by p300 or CBP knockdown. Despite this, iBAT masses were unexpectedly altered after diet in male mice only. Although not examined here, such context-specific changes may reflect different roles of p300 and CBP in regulating the lipid uptake, handling, and/or storage, and not thermogenesis per se given body composition was similar between control and knockouts.

Furthermore, our results suggest that BATokine programming, specifically those important for peripheral glucose handling (42), also remained intact in knockout mice. Consistent with our findings, pan-adipose deletion of either p300 or CBP using *Adipoq*-*Cre* has no effect on body composition and glucose or insulin tolerance (32). However, double knockout of HATs with the *Adipoq*-*Cre* system causes a generalized lipodystrophy phenotype, hyperglycemia, hyperlipidemia, and hepatomegaly (32). Thus, the presence of one HAT in the absence of the other appears to compensate to maintain brown adipose H3K27ac distribution and BAT functioning during diet-induced obesity.

Unlike classical brown fat whose development arises prenatally from *Myf5*+ precursor cells of the dermomyotome, beige adipocytes have a distinctive cellular origin and are induced postnatally by cues like cold exposure, hormones, or even during diseases like cancer cachexia (6). Although p300 and CBP are individually dispensable for tissues of *Myf5*+ origin like skeletal muscle (52, 53) and BAT (shown here), it was unclear whether this was also the case for beige adipocytes in vivo. We chose to examine this pharmacologically by treating animals with the highly selective β_3_-AR agonist CL-316,243 and examining thermogenic markers within inguinal subcutaneous fat, which has a high propensity for browning (6). We found that expression of thermogenic genes, UCP-1 protein, and most mitochondrial respiratory chain components was induced comparably by CL treatment in knockout mice relative to littermate controls. Our results suggest that p300 and CBP alone are not obligatory for beige adipogenesis, which is supported by previous work that cold-induced thermogenic gene expression profiles of inguinal fat are unaltered by pan-adipose p300 deletion (32). Interestingly, pan-adipose CBP deletion does have an effect on thermogenic gene expression (32). Our contrasting results likely reflect the differences in the Cre systems with CBP knockout occurring at different times and in different cell populations. We show that the presence of CBP or p300 specifically in thermogenic adipocytes is sufficient to induce the appearance of beige adipocytes in vivo despite knockout of the other, similar to brown fat.

Brown and beige fats play an integral role in thermoregulation and systemic metabolism in mice. Although a wealth of rodent studies show that enhanced BAT and beige activity protects against obesity, adult humans have relatively little active brown fat (56). Despite this, human BAT activity is inversely correlated with body mass index and fasting blood glucose (13, 57–59). As such, enhancing the functional capacity of brown/beige fat could help mitigate metabolic dysfunction. However, our understanding of molecular factors important for establishing, maintaining, and controlling the adaptability of thermogenic fats is incomplete. Mechanisms governing chromatin state, and therefore gene programming, are integral in this respect. This is the first report examining the requirement of the H3K27 acetyltransferase p300 or CBP within thermogenic adipose tissues specifically. We show here that p300 and CBP are individually dispensable for the formation and activation of brown and beige adipose tissues in vivo, possibly due to functional compensation by the other.

## DATA AVAILABILITY

The data underlying this article will be shared on reasonable request to the corresponding author.

## SUPPLEMENTAL MATERIAL

10.6084/m9.figshare.26207825 Supplemental Figs. S1–S4: https://doi.org/10.6084/m9.figshare.26207825.

## GRANTS

This work was supported by the Canadian Institutes of Health Research (CIHR), Grant PJT-168982 (to W.T.G.); the Canada Research Continuity Emergency Fund (to W.T.G.); and the BC Children’s Hospital Research Institute (BCCHRI), Grant F19-04788 (to W.T.G.). W.T.G. was supported by BCCHRI through an intramural IGAP Award. D.G. was supported by a Michael Smith Health Research BC Fellowship: 18533. M.S.C. was supported by a UBC Faculty of Medicine Graduate Award: 6442 and a CIHR Canada Graduate Scholarship Master’s Award: 6556.

## DISCLOSURES

No conflicts of interest, financial or otherwise, are declared by the authors.

## AUTHOR CONTRIBUTIONS

D.G. and W.T.G. conceived and designed research; D.G. and M.S.C. performed experiments; D.G. and M.S.C. analyzed data; D.G. and M.S.C. interpreted results of experiments; D.G. and M.S.C. prepared figures; D.G. and M.S.C. drafted manuscript; D.G., M.S.C., and W.T.G. edited and revised manuscript; D.G., M.S.C., and W.T.G. approved final version of manuscript.
